# Tissue-specific transcriptomic changes associated with AmBisome® treatment of BALB/c mice with experimental visceral leishmaniasis

**DOI:** 10.12688/wellcomeopenres.15606.1

**Published:** 2019-12-10

**Authors:** Sarah Forrester, Karin Siefert, Helen Ashwin, Najmeeyah Brown, Andrea Zelmar, Sally James, Dimitris Lagos, Jon Timmis, Mitali Chatterjee, Jeremy C. Mottram, Simon L. Croft, Paul M. Kaye

**Affiliations:** 1 York Biomedical Research Institute, University of York, York, YO10 5DD, UK; 2 Department of Immunology and Infection, London School of Hygiene & Tropical Medicine, London, WC1E 7HT, UK; 3 Biosciences Technology Facility, University of York, York, YO10 5DD, UK; 4 Department of Electronic Engineering, University of York, UK, York, YO10 5DD, UK; 5 Department of Pharmacology, Jawaharlal Institute of Post Graduate Medical Education and Research, Kolkata, 700 020, India

**Keywords:** Leishmania, amphotericin B, host response, transcriptomics

## Abstract

**Background:** Liposomal amphotericin B (AmBisome®) as a treatment modality for visceral leishmaniasis (VL) has had significant impact on patient care in some but not all regions where VL is endemic.  As the mode of action of AmBisome® *in vivo *is poorly understood, we compared the tissue-specific transcriptome in drug-treated vs untreated mice with experimental VL.

**Methods: ** BALB/c mice infected with *L. donovani w*ere treated with 8mg/kg AmBisome®, resulting in parasite elimination from liver and spleen over a 7-day period. At day 1 and day 7 post treatment (R _x_+1 and R _x_+7), transcriptomic profiling was performed on spleen and liver tissue from treated and untreated mice and uninfected mice.  BALB/c mice infected with *M. bovis* BCG (an organism resistant to amphotericin B) were analysed to distinguish between direct effects of AmBisome® and those secondary to parasite death.

**Results:** AmBisome® treatment lead to rapid parasitological clearance.  At R _x_+1, spleen and liver displayed only 46 and 88 differentially expressed (DE) genes (P<0.05; 2-fold change) respectively. In liver, significant enrichment was seen for pathways associated with TNF, fatty acids and sterol biosynthesis.  At R _x_+7, the number of DE genes was increased (spleen, 113; liver 400).  In spleen, these included many immune related genes known to be involved in anti-leishmanial immunity. In liver, changes in transcriptome were largely accounted for by loss of granulomas.   PCA analysis indicated that treatment only partially restored homeostasis.  Analysis of BCG-infected mice treated with AmBisome® revealed a pattern of immune modulation mainly targeting macrophage function.

**Conclusions:** Our data indicate that the tissue response to AmBisome® treatment varies between target organs and that full restoration of homeostasis is not achieved at parasitological cure.  The pathways required to restore homeostasis deserve fuller attention, to understand mechanisms associated with treatment failure and relapse and to promote more rapid restoration of immune competence.

## Introduction

Viscerotropic strains of *Leishmania donovani* and *Leishmania infantum*, causing visceral leishmaniasis (VL), are responsible for a significant health burden worldwide, with 200,000 to 400,000 new cases reported annually and ~20,000–40,000 deaths ^1^. One of the principal drugs used in the treatment of VL is amphotericin B, a repurposed anti-fungal agent which has been used to treat VL in multiple formulations, including both free drug, and various liposomal formulations ^2, 3^. AmBisome ^®^, a formulation based on incorporation of amphotericin B into liposomes, is currently the first line therapy for VL in South Asia ^3^ provided by the manufacturer Gilead initially at reduced cost and subsequently as a donation ^4^. The introduction of AmBisome ^®^ monotherapy has contributed significantly to the current elimination effort for VL in South Asia, but its efficacy in East Africa, alone or in combination with other drugs has been disappointing ^5^. Recent studies suggest that the efficacy of AmBisome ^®^ monotherapy in HIV-VL patients can be improved by combination with miltefosine, extending its utility for this indication in East Africa ^6^. AmBisome ^® ^ has also been used for cutaneous leishmaniasis (reviewed in 7).

Amphotericin B is a macrocyclic, polyene antibiotic produced by *Streptomyces nodosus* and it is known to act via irreversibly binding to ergosterol in the cell membrane, causing disruption of membrane integrity and cell death ^8, 9^. Off target toxicity is likely due to binding of cholesterol in mammalian cell membranes and this is minimized by sequestration of amphotericin B within liposomes, such as in AmBisome ^®^^10^. Delivery into cells via liposomes may allow amphotericin to display additional cidal properties including generation of reactive oxygen species, possibly mediated through binding to ergosterol in the membranes of intracellular vesicles or mitochondria ^11^. The immune properties of amphotericin B and its liposomal formulations have been extensively described elsewhere ^11, 12^. For example, free amphotericin B and to a lesser extent liposomal amphotericin B were shown to inhibit T and B cell proliferation *in vitro* ^13^, and *in vitro* and *in vivo* CD8 ^+^ T cell-mediated cytotoxicity ^14^. Both free and liposomal amphotericin B also induce myeloid cell inflammatory cytokine production ^15, 16^, likely via interaction with TLR2 and CD14 ^17^ and this probably underlies many of the immunomodulatory properties observed in different models of fungal immunity ^11^. Less work has been done to unravel the immunomodulatory role of amphotericin B in the context of leishmaniasis. For example, in humans treated with another liposomal formulation Fungisome ^®^, cytokine responses elicited from re-stimulated PBMC one week after single dose treatment were indicative of cure and potential for relapse ^18^. A further study reported altered levels of anti-inflammatory cytokines in BALB/c mice infected with *L. donovani* and treated with Kalsome10, another liposomal formulation ^19^. A comprehensive analysis of host response changes following AmBisome ^®^ treatment using whole blood transcriptomic analysis ^20^ as well as a detailed investigations of CD8 ^+^ and CD4 ^+^. T cell phenotypes pre and post treatment have recently been reported from studies of VL patients in Bihar ^21, 22^. However, studies on systemic responses pre and post treatment are rare and more limited in scope, given the ethical challenges of such work in humans. For example, TLR2 and TLR4 mRNA accumulation was reported to be elevated in pre-treatment splenic aspirates compared to those taken 3–4 weeks after treatment ^23^, as were mRNAs for IL-27p28, EBI-3 and IL-21 ^24^. We have found no reports on changes in the hepatic immune response to *L. donovani* infection in humans.

The spleen and liver are major targets of human infection and at least in rodents, show differential immune and immunopathological responses to *L. donovani* infection ^25– 27^. Furthermore, hepatic and splenic dysfunction account for some of the more serious pathologies associated with VL, including splenomegaly, anaemia, thrombocytopenia, as well clinical characteristics of hepatitis and cirrhosis. Of note, spleen size at discharge was found to be a major risk factor for relapse after AmBisome ^®^ treatment in India ^28^ and after sodium stibogluconate (SSG) or SSG/ paromomycin (PM) treatment in southern Sudan ^29^. In an initial report ^30^, we used transcriptomic profiling to define changes in spleen, liver and blood transcriptome over the course of 42 days of infection in the BALB/c mouse model of VL. Here, we extend this analysis to ask the following questions: i) what are the systemic transcriptional changes that accompany AmBisome ^®^ treatment in this model? and ii) to what extent does the spleen and liver transcriptome return to a homeostatic state following parasitological cure? In addition, we define the extent to which AmBisome ^®^ treatment affects host transcriptome in the absence of microbicidal activity, using a model of *M. bovis* BCG infection. The results reported here indicate that drug treatment leads to a rapid resolution of granulomatous inflammation in the liver which is reflected by whole scale changes in transcriptome associated with loss of immune cells. In contrast, splenomegaly remains after parasitological cure and the transcriptome reflects ongoing processes of tissue remodelling and enhanced stem and hematopoietic activity. Direct effects of AmBisome ^®^ are related to low grade myeloid cell activation. These data provide novel insights into disease resolution after chemotherapy and also highlight potential targets for improving or accelerating a return to homeostasis.

## Methods

### Ethics statement

Experiments were approved by the Animal Welfare and Ethics Review Bodies of the University of York and the LSHTM and the work was performed under UK Home Office license (PPL 60/4377; PPL 70/6997; PPL 70/8207). Mice were killed by exsanguination under terminal anaesthesia prior to tissue collection, as described below.

### Mice and infections

Female BALB/c mice (Charles River, Margate, UK) weighing 20±1 gm and health screened to FELASA 67M standard and maintained under specific pathogen free conditions in individually ventilated cages were used in this study. *Leishmania donovani* (LV9; WHO Ref name: MHOM/ET/67/HU3)) parasites were maintained in B6. *Rag1* ^-/-^ mice and amastigotes prepared following tissue disruption and differential centrifugation, as described elsewhere ^31^. 2×10 ^7^ amastigotes in 150 μl RPMI were injected intravenously (i.v.) via the lateral tail vein and without anaesthetic to initiate infection. After infection, mice were allocated to cages of 5 and provided food and water ad libitum. The naïve and d36 and d42 untreated infected mice reported here (n=5 per group) were part of the previously described CRACK-IT_2 cohort ^30^. An additional 10 mice were infected at the same time and following randomisation along with the other groups, treated at d35 with 8mg/kg AmBisome ^®^ i.v. Five mice from this cohort were killed at day 36 (R _x_+1) and day 42 (R _x_+7) and compared to their time matched counterparts. All animals were killed and processed over an approximate time period of 4–6h beginning in the morning. Tissues were aseptically removed post mortem and stored/processed as detailed below. Parasite loads were determined using the impression smear technique and are reported as Leishman Donovan Units ^32^. All downstream tissue analysis was performed blinded to group by investigators not involved in animal handling. For infection with *M. bovis* BCG, ten additional female BALB/c mice were infected i.v. with 2x10 ^6^ CFU / mouse of the BCG-SSI (Aeras stock Lot No. 050613MF) and at 35d post infection, five were treated with AmBisome ^®^ as above and five remained untreated. All BCG-infected mice were killed at d42 (R _x_+7) and tissue homogenates were plated for 22 days. Mycobacterial counts are expressed as log CFU/organ.

### Tissue transcriptomics

Transcriptomic analysis of RNA using Agilent SurePrint G3 mouse GE 8x60 microarray chips isolated from tissues of *Leishmania* infected mice were processed and analysed as described in 30. Tissue from mice infected with *M. bovis* BCG, were processed for transcriptomic analysis by RNA-Seq. Paired end illumina RNA sequencing libraries were generated using polyA enrichment followed by using the NEB next RNA kit as per manufacturer’s instructions. Libraries were sequenced by Illumina HiSeq 2500, using 2 x 100bp reads. Universal illumina adaptors were removed using cutadapt v 1.8.3. Unpaired reads were removed, and reads were further trimmed and scored for quality using sickle v 1.33 ( https://codeload.github.com/najoshi/sickle/tar.gz/v1.33). Reads were then aligned using STAR aligner v 2.5.1b using the sjdbOverhang 150 to Mus Musculus version 89 from ENSEMBL ^33, 34^. Non uniquely reads were filtered out and gene expression was calculated using the union algorithm in HTseq-count. DE expressed genes were identified through the R package edgeR ^34^. The data was normalised using this package, and pairwise comparisons were performed using a generalised linear model and using Bonferonni multiple testing threshold of 0.05. Unexpressed and lowly expressed transcripts were removed using the logged counts per million values of expression, and by removing those in the bottom 20 percentile. The prcomp function in R was used to generate eigenvalues. The first two principal components were used to look at groupwise variation, which accounted for >80% of the variation between the groups. Volcano plots were generated to look at pairwise DE, using log2FC that were calculated from the mean values of each group in the pairwise comparison. EnrichR was also used to perform additional enrichment analysis ^35, 36^ and DE genes were assigned into KEGG groups using the R package ^37^. Adjusted p values are shown in text.

### Quantitative morphometry

Digital whole slide images of infected livers were prepared from F4/80 and DAPI stained cryosections using an AxioScan Z1 slide scanner (Zeiss, Jena, Germany) and analysed using a bespoke macro developed within the Strataquest image analysis software package (TissueGnostics, Vienna, Austria) as described previously ^30^. Data are shown for the number of granulomas per mm ^2^ and the size of individual granulomas.

## Results

### Transcriptomic response to treatment of *L. donovani* infection with AmBisome ^®^

**Figure 1.**
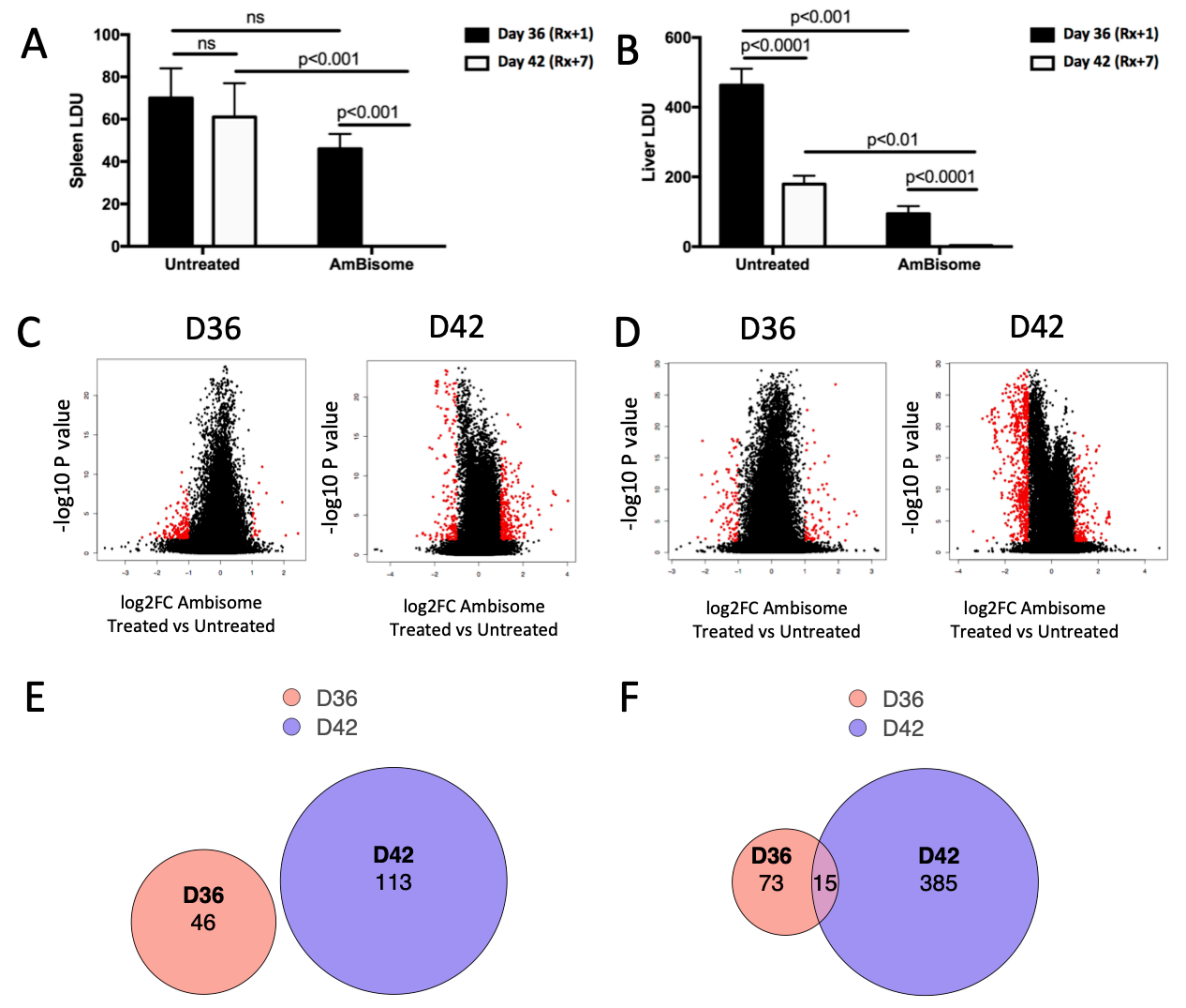
Transcriptional changes associated with AmBisome ^®^ treatment in *L. donovani*-infected BALB/c mice. Cohorts of weight matched female BALB/c mice were infected with ~2×10 ^7^ *L. donovani* amastigotes and at day 35 randomised to receive AmBisome (8mg/kg) or saline (untreated). **A** and **B**. Parasite loads were determined in spleen ( **A**) and liver ( **B**) at day 36 (R _x_+1) and day 42 (R _x_+7). Data are shown as mean Leishman Donovan Units ± SEM (n=5 per group), with p values as indicated. **C** and **D**. Differentially expressed genes in spleen ( **C**) and liver ( **D**) are presented using a volcano plot, with red symbols indicating genes passing the FDR<0.05 and log2FC=1 cut off. Genes with positive DE are UP in AmBisome ^®^-treated vs. untreated mice and vice versa. **E** and **F**. Venn diagrams to indicate the number of up and down regulated genes and their overlap in spleen ( **E**) and liver ( **F**), comparing AmBisome ^®^-treated vs. untreated mice.

To study the impact of AmBisome ^®^ treatment on host transcriptional profile, we infected BALB/c mice for 36 days, a time point when pronounced splenomegaly and a stable splenic parasite burden is established. At this time, hepatic resistance to infection is already coming into play, with the development of a florid granulomatous response, whereas the spleen is undergoing extensive remodelling due to chronic inflammation. These histopathological changes are accompanied by significant changes to the host transcriptome ^30^. On day one following single dose administration of AmBisome ^®^ (R _x_+1) there was no significant effect on splenic parasite burden ( Figure 1A) or spleen weight (786 ± 22 vs. 822 ± 50 mg for untreated and AmBisome ^® ^treated mice, respectively). By seven days post treatment (R _x_+7), complete clearance of parasite load to below detection limits of the impression smear technique had occurred ( Figure 1A) and spleen weight was reduced by approximately 33% (784 ± 35 vs. 526 ± 50; p<0.01). In the liver, however, AmBisome ^®^ had a more rapid leishmanicidal effect, reducing parasite load by ~80% at R _x_+1 and again leading to complete clearance by R _x_+7 ( Figure 1B). Hepatomegaly was not significantly different at R _x_+1 (1592 ± 58 vs. 1632 ± 26 mg) whereas by R _x_+7, there was a decrease in liver size of approximately 16% (1606 ± 65 vs. 1348 ± 56 mg; p<0.05). Hence, as previously shown, AmBisome ^®^ is highly effective in treating experimental VL in BALB/c mice ^38^.

To evaluate how this pattern of cure was reflected in host response, we studied spleen and liver tissue transcriptomes at R _x_+1 and R _x_+7, making comparisons against untreated mice at each time point, to reduce possible false positives due to any changes in transcriptomic response due to the natural progression of infection. Expression data are shown as volcano plots of log2FC against p value for AmBisome ^®^ treated vs. untreated mice for spleen ( Figure 1C) and liver ( Figure 1D) at both R _x_+1 and R _x_+7. Differentially expressed (DE) genes passing the cut-off threshold of FDR<0.05 and log2FC≥1 are shown in red. The distribution of genes passing this threshold in spleen and liver at each time are also indicated by venn diagrams ( Figure 1E and F).

At day R _x_+1 in the spleen, only 46 genes (5 UP and 41 DOWN) were DE between AmBisome ^®^-treated and untreated mice at day R _x_+1 ( Figure 1C and E), compared to 88 DE genes (39 UP and 49 DOWN) in the liver ( Figure 1D and F; *Extended data:* Table S1), with no overlap between gene lists in the two tissues. Using the protein-protein interaction database STRING ^39^, the products of down regulated genes in the spleen showed no specific enrichment. In the liver, up-regulated genes were enriched for GO terms “metallopeptidase activity” (FDR 0.0156), “cellular response to interleukin 1” (FDR 0.0225), collagen catabolic process and cellular response to tumour necrosis factor (both FDR 0.0258). Down-regulated hepatic genes were enriched for KEGG pathways linoleic acid metabolism (FDR 0.0111), bile secretion (FDR 0.0167), and steroid hormone biosynthesis and retinol metabolism (all FDR 0.0173).

At R _x_+7, 113 genes were DE in spleen (58 UP and 55 DOWN; Figure 1E; *Extended data:* Table S1). No specific enrichments were found using STRING within the upregulated gene list, whereas down regulated genes were significantly enriched for GO terms “response to molecule of bacterial origin”, “defence response”, “inflammatory response” and “cellular response to IL-1” (all FDR 5.62x10 ^-4^). Enriched KEGG pathways included “TNF signalling” (FDR 4.37x10 ^-5^), “Salmonella infection” (FDR 0.00407), “Chagas disease” (FDR 0.0078) and “Leishmaniasis” (FDR 0.0205). Immune-related molecules down regulated in expression in the spleen at R _x_+7 included *Ccl2*, *Cxcl2*, *Irg1*, *Il6*, *IL23r*, *Msr1*, *Nos2*, *Ptgs2, Saa1, Saa3*, *Timp1* and *Ms4a6d.* Surprisingly, no other cytokines, TNF family members or molecules with CD designations were affected by AmBisome ^®^ treatment in the spleen, despite parasite clearance.

**Figure 2.**
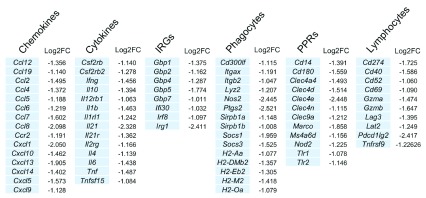
Immune genes down regulated following AmBisome ^®^ treatment in the liver. 74 immune-associated down regulated genes grouped according to functional category or main cellular localisation. Log2 fold change in expression is indicated. IRGs, interferon regulated genes, PPRs, pattern recognition receptors. Full list of up and down regulated genes at each time post treatment are found in *Extended data:* Table S1.

In liver, 400 genes were DE by R _x_+7 (319 DOWN, 81 UP; Figure 1F; *Extended data:* Table S1). Similar to spleen, down-regulated genes were significantly enriched for GO terms including “defense response”(FDR 2.77x10 ^-39^) and immune system process” (FDR 1.37x10 ^-36^) and KEGG pathways “cytokine-cytokine receptor interaction” (FDR 1.15x10 ^-14^) and “leishmaniasis” (FDR 1.55x10 ^-13^) forming a major cluster of interaction defined using STRING ( *Extended data:* Figure S1). Genes clearly associated with immune function (e.g. CD designation, cytokines/chemokines, lectin-like receptors etc) accounted for 23% (74/319) of these down-regulated genes and featured a number of genes associated with pathogen recognition, macrophage activation and T cell regulation, including the cytokines *Tnf* and *Ifng*, and the checkpoint regulators *Lag3*, *Cd274* (PD-L1) and *Pdcd1lg2* (PD-L2) ( Figure 2). In addition, a large cluster of genes associated with cell cycle regulation was also down regulated ( *Extended data:* Figure S1). In contrast, up-regulated genes were enriched for GO terms related to fatty acid metabolic processes” (FDR 5.03x10 ^-5^), monocarboxylic acid metabolic process (FDR 1.0x10 ^-4^), “lipid metabolic processes” (FDR 1.17x10 ^-4^) and KEGG pathways “fatty acid elongation”, “biosynthesis of unsaturated fatty acids” and insulin signalling pathway (all FDR 0.0109) ( *Extended data:* Figure S2).

Only a small subset of DE genes was co-regulated in both spleen and liver at R _x_+7 (6 UP and 24 DOWN; *Extended data:* Table S1). Commonly upregulated genes were *Ccrl1/Ackr4* (atypical chemokine receptor 4), *Epx* (eosinophil peroxidase), *Prg2* (proteoglycan 2 / mMBP-1), *Prg3* (proteoglycan 3 / MBP2), *Ear7* (eosinophil-associated, ribonuclease A family, member 7) and *Ear6* (eosinophil-associated, ribonuclease A family, member 6), suggesting a heightened eosinophilic response in both tissues after treatment with AmBisome ^®^. Within the common downregulated gene list, there was enrichment for GO terms related to host defence and inflammation (GO:0009617, FDR 6.70x10 ^-6^; GO:0006954, FDR 1.33x10 ^-5^; GO:0006952, FDR 3.39x10 ^-5^) and this list also included markers related to macrophage activation including *Pdcd1lg2* (PD-L2), *Irg1*, *Fos*, *Marco*, *Nos2*, *Ptgs2*, *Ccl2*, *Clec4e*, *Ms4a6d*, and *Il6*.

Collectively, these data indicate that although both spleen and liver ultimately clear their parasite burden by R _x_+7, they do so with differing kinetics and with tissue-specific changes in host transcriptional profile.

### Analysis of immune pathways affected by AmBisome ^®^ treatment

**Figure 3.**
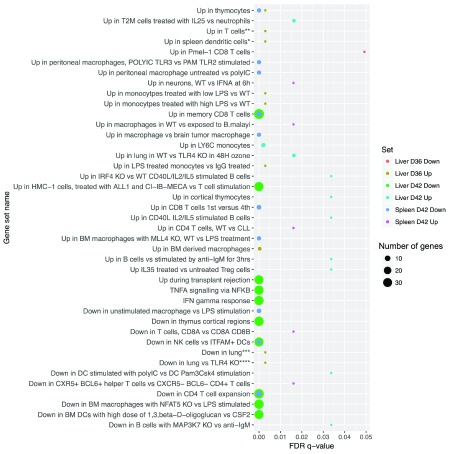
GSEA analysis of DE genes in spleen and liver after AmBisome ^®^ treatment. Figure shows pathway enrichment for DE genes identified in *Extended data:* Table S1, according to gene set and number of genes. Full descriptions of gene sets and analysis are provided in *Extended data:* Table S2.

In order to gain a deeper appreciation of immune-related changes associated with AmBisome ^®^ treatment, gene set enrichment analysis was performed. For each DE gene list generated in spleen and liver at both R _x_+1 and R _x_+7, we conducted pathway enrichment against the MSigDB Hallmark gene set panel and the Immunology C7 panel. Full outputs from the analysis are included in the *Extended data* (Table S2). The top ten most significant enrichments (above a threshold of FDR 0.05) are shown for each time point and each direction ( Figure 3). Genes downregulated in AmBisome ^®^ treated mice at R _x_+7, most prominently within the liver, were significantly enriched in MSigDB gene sets associated with lymphocyte and myeloid cell responses, notably those relating to T cell activation and IFNγ and TNF responses signalling. Collectively, these data suggest that by R _x_+7, there has been a significant reduction in T cell and myeloid cell content and/or function in both spleen and liver.

### Reduction in granulomatous inflammation is largely responsible for observed changes in hepatic transcriptome following AmBisome ^®^ treatment

**Figure 4.**
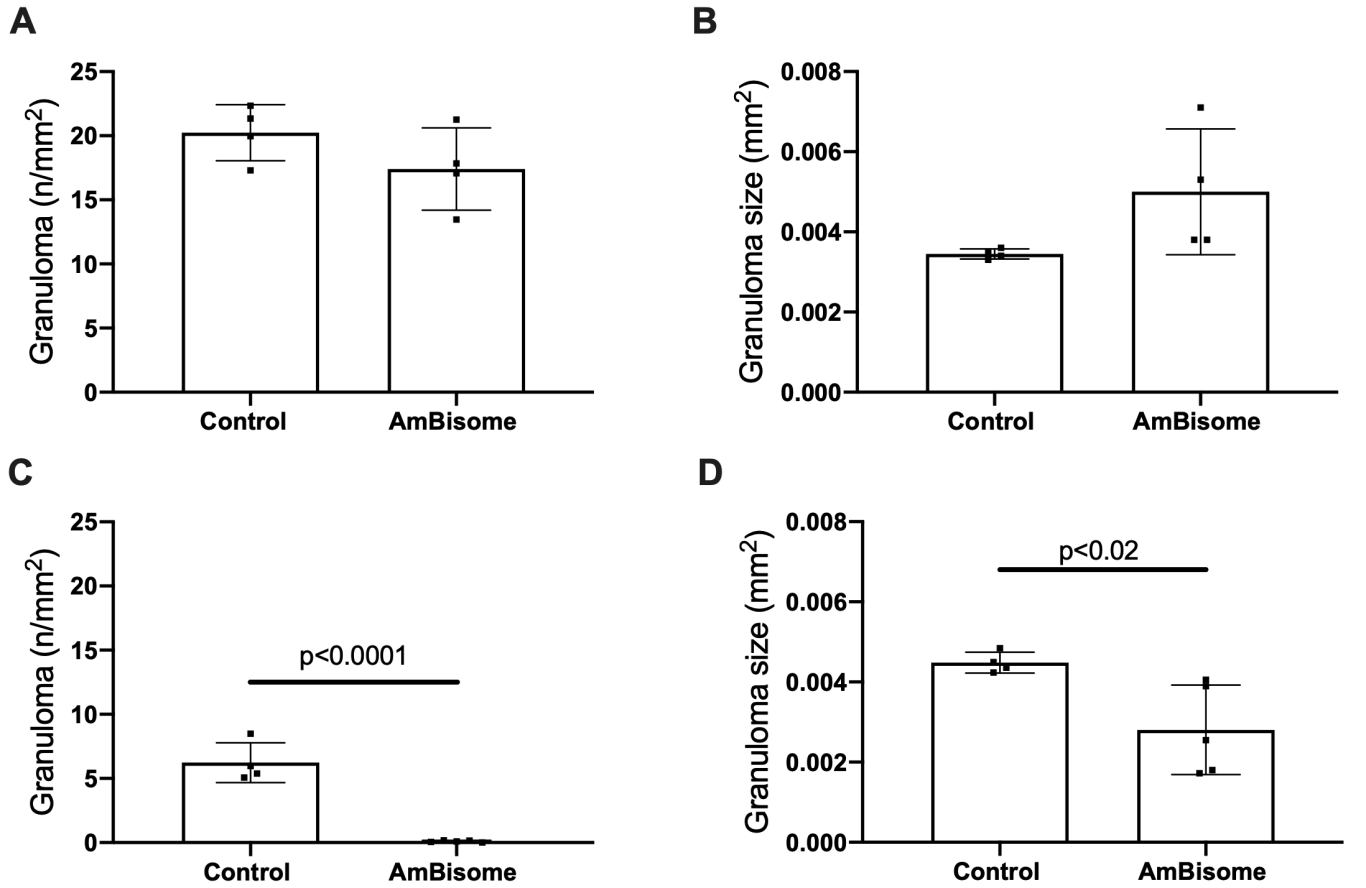
Granulomatous inflammation in *L. donovani*-infected BALB/c mice treated with AmBisome ^®^. The hepatic granulomatous response was evaluated using quantitative morphometrics on F4/80-stained cryosections, as Methods. **A** and **B**. Response to treatment at R _x_+1. **C** and **D**. Response to treatment at R _x_+7. Data are presented as number of granulomas per unit area ( **A** and **C**) and average granuloma size ( **B** and **D**). Data are derived from whole mount analysis of a single section per mouse and n=4 or 5 mice per group, with p values as indicated determined by unpaired t test.

To determine whether the changes in hepatic transcriptome associated with AmBisome ^®^ treatment were a result of changes in gene expression or in the cellular composition of the tissue, we analysed tissue sections to describe the extent of granulomatous inflammation, a hallmark of the hepatic response to *L. donovani* ^26^. We used quantitative image analysis based on granulomas defined by a F4/80 ^+^ core and surrounded by a cellular infiltrate (see Methods; Figure 4). At R _x_+1, we observed no significant change in granuloma number or granuloma size compared to non-drug treated mice ( Figure 4A and B). Although granuloma numbers were already declining in infected untreated mice (compare Figure 4A and C), commensurate with the known kinetics of the hepatic response ^32^, AmBisome ^®^ treatment rapidly accelerated granuloma clearance from the liver, with a >99% reduction in granuloma number per unit area (6.23±1.53 vs. 0.098±0.07/mm ^2^, in AmBisome ^®^ treated vs. untreated mice respectively; Figure 4C) at R _x_+7. Of interest, the few remaining granulomas were also significantly smaller in size ( Figure 4D). Taken together, it appears that the changes in transcriptomic profile associated with AmBisome ^®^ treatment at the tissue level reflect changes in cellularity and a cessation of granulomatous inflammation following parasite clearance, rather than changes in cellular gene expression *per se*.

### AmBisome ^®^ has subtle direct effects on the host transcriptome masked by its anti-leishmanial properties

**Figure 5.**
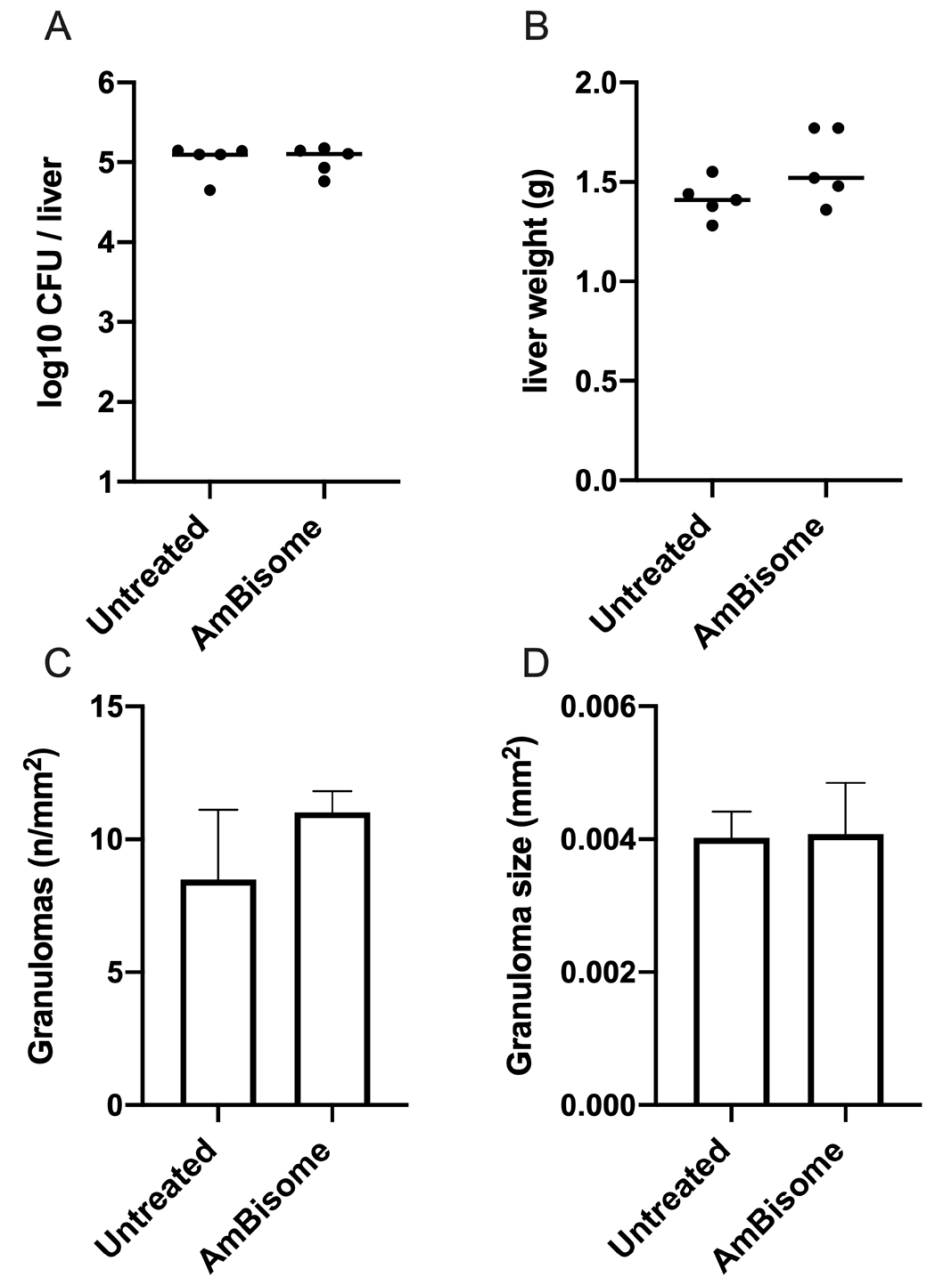
AmBisome ^®^ treatment of *M. bovis* BCG-infected BALB/c mice does not impact on hepatic mycobacterial load, hepatomegaly or granulomatous inflammation. Female BALB/c mice were infected with ~2×10 ^6^ M. bovis BCG (BCG-SSI, Aeras) and at d35 post infection treated or not with 8mg/kg AmBisome ^®^. At R _x_+7, mice were killed, livers weighed, and liver tissue plated to assess growth of viable mycobacteria and processed for histology. **A**. hepatic mycobacterial tissue load. **B**. Hepatomegaly. **C** and **D**. Granulomatous inflammation was quantified by number of granulomas per unit area ( **C**) and average granuloma size ( **D**). Data are presented for individual mice and the mean is shown (bar); p=ns for CFU/organ and liver weight (n=5 per group), and for measures of granulomatous inflammation (n=4 per group).

Anti-leishmanial drugs, including AmBisome ^®^, have been ascribed immunomodulatory properties ^11^, though limited studies have addressed this question *in vivo*. We therefore asked whether the clear changes in inflammatory response associated with the leishmanicidal activity of AmBisome ^®^ might obscure any direct effects of the drug on host cellular function. In order to remove this confounder, we made use of the observation that *M. bovis* BCG also induces hepatic granulomas ^40^, yet this organism has not been reported to be sensitive to amphotericin B. We infected a cohort of BALB/c mice with *M. bovis* BCG and at d35 post infection, mice were either treated with AmBisome ^®^ or remained untreated. CFU counts from liver at R _x_+7 confirmed that *M. bovis* BCG was insensitive to AmBisome ^®^ ( Figure 5A). There was also no reduction in hepatomegaly (1412 ± 98 vs. 1580 ± 183 mg in untreated and treated mice, respectively; Figure 5B). Furthermore, histological analysis indicated that AmBisome ^®^ treatment did not affect granuloma number or cellularity in *M. bovis* BCG infected mice ( Figure 5C and Figure 5D). Transcriptomic profiling was performed, and DE genes identified by comparison of untreated and AmBisome ^®^-treated *M.bovis* BCG infected mice at R _x_+7.

Only 3 genes passed a threshold of logCPM >1 and log2FC > 1 in the liver of *M.bovis* BCG-treated mice at R _x_+7 (UP: *Lcn2, Mmp12,* and *Orm2*). To explore whether more subtle effects could be detected, we removed the threshold and examined all genes where there was statistical significance of P<0.05. 295 genes (208 UP and 87 DOWN) were scored as DE on this criteria and pathway analysis identified a high degree of enrichment for genes associated with macrophage activation ( Table 1; *Extended data:* Table S3). Importantly, genes associated with phagocytes and innate recognition that were identified as down regulated in *L. donovani*-infected treated mice were found to be upregulated in similarly treated *M.bovis* BCG-treated mice, including *Tlr2*, *Sirpa*, *Nos2*, *Ms4a6d*, *Lyz2*, *Irf8* and *H2-Aa*. This pattern of a low-level change in expression of myeloid-associated genes was also evident more broadly, with high representation within gene sets identified in relation to myeloid function in a range of diseases (FDR values of 2.96 x10 ^-10^ to 5.33x10 ^-13^; *Extended data:* Figure S3). In contrast, genes associated with lymphocyte function and related cytokine and chemokine genes whose mRNAs were reduced in abundance after treatment of *L. donovani* infected mice ( Figure 2), were unaltered in treated *M. bovis* BCG-infected mice ( *Extended data:* Table S2). Collectively, these data suggest AmBisome ^®^ treatment stimulates low grade activation of myeloid cell function, but that this may be masked due to the much more significant negative effects associated with loss of inflammatory stimulus resulting from the elimination of *Leishmania* amastigotes. Hence, the main impact of AmBisome ^®^ on the liver transcriptome in *L. donovani*-infected mice as seen here likely represents an indirect consequence of its leishmanicidal activity and reduction in parasite load.

**Table 1.** Gene set enrichment analysis of DE genes after AmBisome ^®^ treatment of *M. bovis* BCG-infected BALB/c mice.

Mouse Gene Atlas					
Term	Overlap	P-value	Adjusted P-value	Odds ratio	Combined Score
liver	52/928	2.37E-17	2.28E-15	3.891	148.96
macrophage_peri_LPS_thio_0hrs	28/353	2.72E-13	1.31E-11	5.508	159.37
macrophage_peri_LPS_thio_1hrs	35/598	1.93E-12	6.18E-11	4.064	109.63
macrophage_peri_LPS_thio_7hrs	33/707	3.00E-09	7.19E-08	3.241	63.61
macrophage_bone_marrow_24h_LPS	23/551	5.47E-06	1.05E-04	2.899	35.12
macrophage_bone_marrow_6hr_LPS	26/730	2.20E-05	3.52E-04	2.473	26.53
dendritic_cells_lymphoid_CD8a+	9/142	2.21E-04	0.0030295	4.401	37.05
spleen	6/100	0.0032574	0.0390883	4.167	23.86
macrophage_bone_marrow_0hr	8/180	0.0046569	0.0496739	3.086	16.57
KEGG 2019 Mouse					
Term	Overlap	P-value	Adjusted P-value	Odds Ratio	Combined Score
Leishmaniasis	11/67	2.87E-09	8.69E-07	11.401	224.26
Tuberculosis	16/178	6.75E-09	1.02E-06	6.2422	117.44
Phagosome	15/180	5.57E-08	5.62E-06	5.7870	96.66
Glycine, serine/threonine metabolism	8/40	8.65E-08	6.55E-06	13.889	225.88
Proteoglycans in cancer	14/203	1.55E-06	9.38E-05	4.7893	64.07
Osteoclast differentiation	11/128	2.50E-06	1.26E-04	5.9679	76.98
Leukocyte transendothelial migration	10/115	6.46E-06	2.80E-04	6.0386	72.16
Hematopoietic cell lineage	9/94	8.57E-06	3.25E-04	6.6489	77.57
Lysosome	10/124	1.26E-05	4.25E-04	5.6004	63.17

Top ten enrichments are shown. Full details of all enrichments and genes are provided in *Extended data:* Table S3.

### Parasitological clearance does not restore immune homeostasis

**Figure 6.**
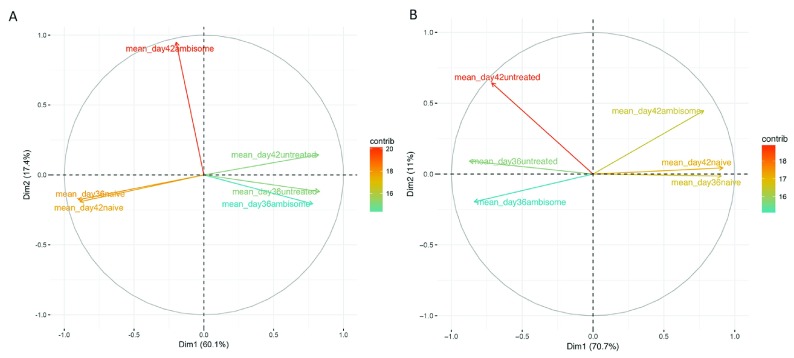
Principal component analysis comparing *L. donovani* infected, AmBisome ^®^-treated *L. donovani*-infected and naïve mice BALB/c mice. **A** and **B**. Eigen values were generated using the normalised expression values for differentially expressed genes in spleen ( **A**) and liver ( **B**). PCA analysis was performed in R using the prcomp package and the percentage contribution each condition (treatment and infection status) has to the expression value/ eigenvalue used to summarise the data; and the directionality of the first two principle components is shown as a biplot. The percentage of the variability in the data explained by first two components is the sum of the percentages given on both the x and y axis.

Although AmBisome ^®^ is highly effective in clearing parasites in animal models and in humans, the extent to which the immune response and other parameters return to homeostasis early after treatment in systemic tissues is unknown. Splenomegaly was clearly still evident at the end of treatment (526 ± 50 vs. 94 ± 7 mg for AmBisome ^®^-treated mice at R _x_+7 and naïve control mice, respectively; p<0.0001), as was hepatomegaly (1348 ± 56 mg vs. 968 ± 49 mg; p<0.001). We therefore compared the transcriptomic profile of untreated and drug-treated infected mice at both R _x_+1 and R _x_+7 with that of age/sex-matched control mice using principal component analysis (PCA), a statistical technique to reduce data dimensionality and identify patterns in data. Only the first two principal components were used to calculate the similarity between groups, as this represented 77.5% and 80.7% of the total variation in the spleen and liver, respectively. The directionality of these is shown as a biplot in Figure 6. In both spleen ( Figure 6A) and liver ( Figure 6B), control naive mice clustered most tightly indicative of a low level of intra-group variability, with the main source of variation along the x axis. Not unsurprisingly, untreated infected mice were most dissimilar to naïve mice and also clustered together. In keeping with the number of DE genes and the kinetics of parasite clearance, untreated and R _x_+1 were more closely aligned than samples from each tissue at R _x_+7. For liver samples, the untreated control mice at day 42 also showed a shift in position compared to d36 untreated mice, indicative of the onset of natural immunity in the liver but not spleen of *L. donovani*-infected mice ( Figure 1). More importantly, R _x_+ 7 samples shifted away from untreated mice and in the direction of naïve mice. However, it was clear that such samples took an intermediate position relative to naïve and untreated infected mice. Based on this criterion, restoration to homeostasis appeared more complete in liver compared to spleen.

### Genes not reverting to baseline expression after AmBisome ^®^ induced parasite clearance

**Figure 7.**
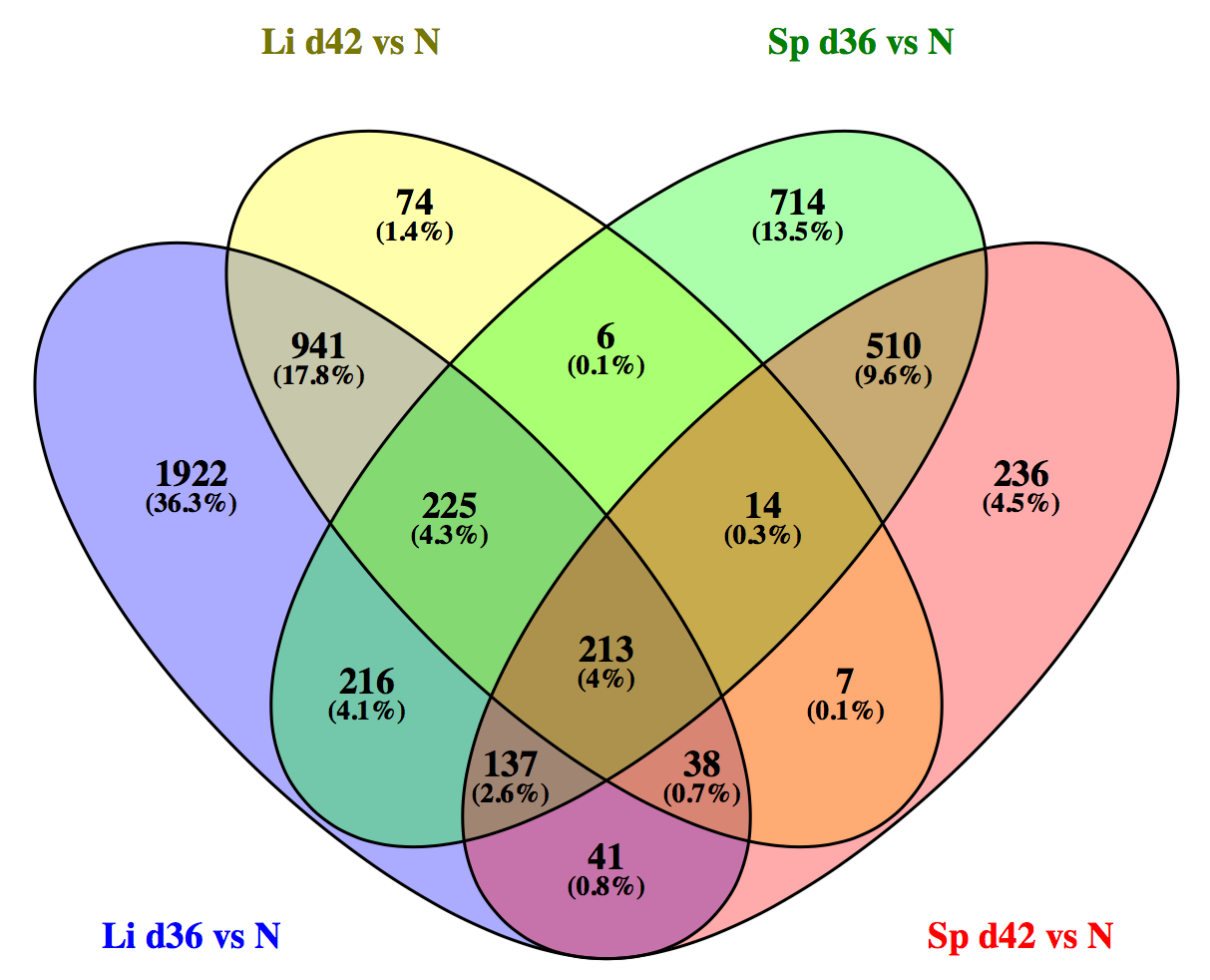
Venn diagram to illustrate overlap in DE genes between all treatment groups. Venn diagram representing all DE genes listed in *Extended data:* Table S4 in spleen and liver at each time point after AmBisome ^®^ treatment.

In the liver, 3733 genes (2107 UP; 1626 DOWN) were DE between AmBisome ^®^-treated and naïve mice at R _x_+1 reducing to 1518 genes (1294 UP; 224 DOWN) at R _x_+7. In the spleen, 2035 genes (881 UP: 1154 DOWN) were DE between treated and naïve mice at R _x_+1 reducing to 1196 genes (481 UP; 715 DOWN) at R _x_+7 ( *Extended data:* Table S4). The overlap between these genes across organ and time post treatment is shown as a venn diagram generated using Venny 2.1 ( Figure 7 ^41^), illustrating the distinctive nature of the return to homeostasis in spleen and liver. Our previous analysis identified 3055 and 2805 DE genes at d42 post infection in liver and spleen respectively, when comparing d42 infected mice with control uninfected mice ^30^. Of the genes DE in the liver between naïve and R _x_+7 mice, 97% (1476/1518) were represented in the original infection-associated gene list. Similarly, 91% (1085/1196) of DE genes in spleen identified in a comparison of R _x_+7 vs naïve mice were also found in the original infected vs naïve DE list. This suggests that most transcriptomic changes that remain after treatment with AmBisome ^®^ represent a subset of those initially induced by infection. However, amongst the 74 immune-associated genes downregulated by AmBisome ^®^ treatment in the liver ( Figure 2), only 9.45% (7/74) returned fully to homeostatic levels ( *H2-M2*, *H2-Eb2*, *Cxcl1*, IL6, *Lag 3*, *Tnfsf15* and *Tnfrsf9* (CD137, 4–1BB), indicating a high level of residual immune activity in the liver despite the loss of discrete granulomas.

In order to further analyse DE genes that did not revert to homeostatic levels, we used gene set enrichment, employing the EnrichR tool. Analysis of the residual 1518 DE genes in the liver post treatment using Go Biological Processes, GO Molecular Function, Mouse Gene Atlas and Wiki Pathways are provided in *Extended data:* Table S5. GO terms related to cytokine signalling, inflammation, T cell and neutrophil mediated immunity, were the most significant. Wiki Pathways identified chemokine signalling, microglia pathogen phagocytosis, IFNγ signalling and TYROBP Causal Network WP4625 (a microglial activation pathway), and the Mouse Atlas analysis revealed predominantly myeloid cell populations. Similar analysis for the residual 1196 DE genes in spleen ( *Extended data:* Table S6) suggested a prominent role for neutrophils and endothelial cells in the post cure phase of experimental VL, including GO Molecular Function terms related to neutrophil mediated immunity, degranulation and activation and both neutrophil and endothelial cell chemotaxis. Similarly, GO Molecular Function terms for a variety of serine-type and metallopeptidases were highly ranked alongside multiple terms related to chemokine biology.

Of note, the top upstream transcription factors predicted by IPA analysis to regulate spleen residual genes were *Gata2* (94 predicted targets; p=3.07x10 ^-43^; z score 1.707, predicting activation of the regulator), *Mrtfb* ( *Mkl2*; 41 predicted targets; p=6.92x10 ^-33^; z score -5.204, predicting inhibition of the regulator) and *Mrtfa* ( *Mkl1*; 49 predicted targets; p=5.12x10 ^-31^; z score -4.232, predicting inhibition of the regulator). *Gata2* is associated with haematopoiesis / stem cell function, whereas *Mrtfb* and *Mrtfa* are associated with tissue organization, abnormal morphology and tissue remodeling, as well as with thrombocytopenia (a hallmark of human and experimental VL ^42, 43^. Collectively, these data suggest that spleen is in a state of active “repair”.

### AmBisome ^®^ treatment is reflected by a loss of a systemic gene signature associated with *L. donovani* infection in mice

**Figure 8.**
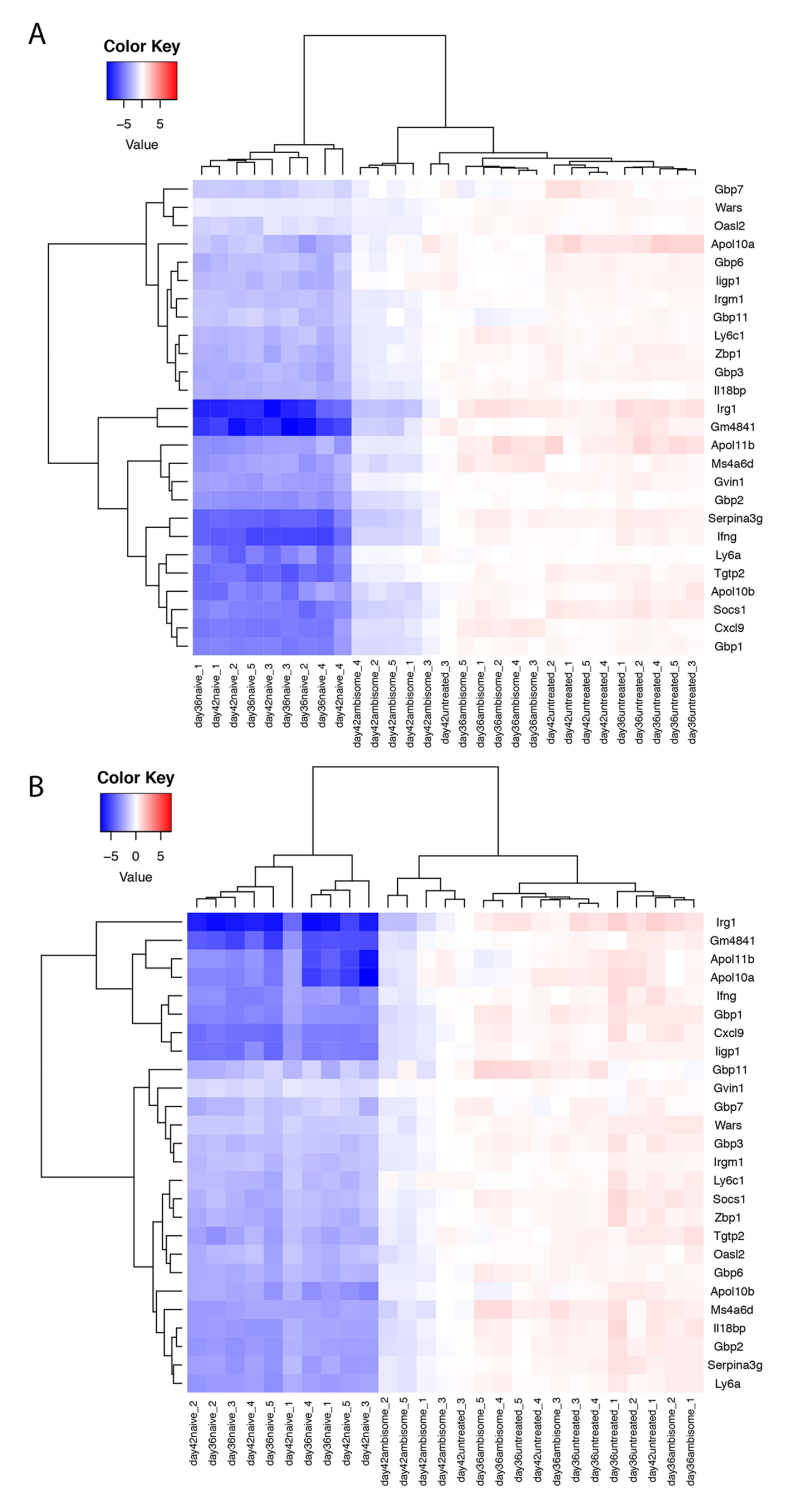
Restoration of 26 gene signature in spleen and liver following AmBisome treatment in *L. donovani* infected BALB/c mice. Data is shown in heat map format for the 26 genes identified as a common transcriptional signature of infection in blood, spleen and liver of *L. donovani* infected mice ^30^. Scale represents gene expression values, with no column wise or row wise normalisation applied. **A** and **B**. The dendrogram illustrates liver ( **A**) and spleen ( **B**) data clustered both by the similarity in the pattern of expression per gene (y axis), and by the similarity in gene expression between individuals (x axis).

Finally, we had previously identified a transcriptomic signature of 26 predominantly interferon regulated genes that was commonly upregulated by infection with *L. donovani* in spleen, liver and blood regardless of time post infection ^30^. We therefore examined the impact of AmBisome ^®^ treatment on expression of this systemic disease-associated signature. In the liver ( Figure 8A) and spleen ( Figure 8B) of AmBisome ^®^-treated mice, the entire signature gene set was downregulated compared to untreated mice, in a time dependent manner. However, a full restoration to the homeostatic mRNA abundance seen in uninfected control mice was only occasionally noted.

## Discussion

VL is a systemic disease and understanding disease progression and the response to chemotherapy is dependent upon a more complete understanding of how target tissues respond over time. Although in recent years the evaluation of immune and other changes associated with human disease progression and drug response has adopted a more systematic approach employing -omics technologies, the invasive nature of tissue sampling has largely restricted this approach to whole blood ^20, 21, 44, 45^. Here, we used the BALB/c model of VL to directly explore the breadth of transcriptomic changes associated with AmBisome ^®^ treatment. Although there are undoubtedly differences between rodent models and humans, as we have recently highlighted ^30^, it is worth recalling the instrumental role that the BALB/c mouse model played in supporting the clinical use of AmBisome ^®^^46, 47^. To study drug response, we chose to focus on a relatively late time (d35 post infection) for drug treatment when chronic pathology is established in the spleen, and used a dose of AmBisome ^®^ for which anti-parasitic efficacy has been shown previously in liver and spleen ^38, 48^.

The hepatic response to AmBisome ^®^ treatment was as expected rapid, with hepatic parasite load reduced by >50% at R _x_+1 and to below detection levels by R _x_+7. At R _x_+1, there appeared no direct histopathological correlate of drug action, but by R _x_+7 most granulomas had regressed, and those that remained had reduced cellularity. Transcriptomic analysis of hepatic tissues largely mirrored this histopathological evaluation. Less than 3% of genes DE during infection ^30^ changed in their mRNA abundance one day after treatment, reflecting the intact granulomatous response. At R _x_+7, when mice had fully cleared their infection to below detection limits, this rose to ~13% (400/3055) of the total infection-induced DE genes, coincident with a loss of granulomas from the tissue. Although pathway and enrichment analysis identified some changes in inflammatory pathways, metalloprotease activity and some metabolic pathways, FDR values were modest at R _x_+7. Such analysis more robustly identified pathways associated with immune function, particularly within the “down regulated” genes, as might be expected from the dramatic loss of inflammatory granulomas. Although by d42 post infection, spontaneous resolution of hepatic infection is already well under way, the reduction of parasite load due to AmBisome ^®^ treatment clearly accelerates this process. Hence, it is not possible to say whether specific cell populations involved in granuloma development and function specifically alter their gene expression profile following treatment. Similarly, it is possible that small changes in parenchymal cell responses after treatment may have been overlooked due to the high signal to noise for immune cells generated by altered states of inflammation. Further studies using spatially resolved and/or single cell analysis will be required to address this question.

To specifically address whether AmBisome ^®^ treatment might impact directly on host response in the absence of any anti-microbial activity we sought an alternate *in vivo* approach. Although AmBisome ^®^ treatment of naïve mice might appear an obvious experimental approach, we felt that this would not fully reflect the potential for drug action on cells already activated during inflammation and through antigen-specific immune responses, nor would it mimic the impact of infection-associated pathology on drug distribution ^38^. We therefore elected to use an infection model with *M. bovis* BCG, which has previously been shown to induce both splenomegaly and granulomatous inflammation in the liver, not too dissimilar from that seen in experimental VL ^26, 40^. At a conventional threshold of 2-fold change, only three genes were found to be DE seven days after AmBisome ^®^ treatment. Removal of this threshold revealed that many of the immune changes observed in *Leishmania*-infected mice treated with AmBisome ^®^ had not occurred in BCG-infected drug treated mice, in keeping with maintenance of granuloma integrity. More strikingly, AmBisome ^®^ treatment of BCG-infected mice uncovered a number of significant albeit small changes in mRNA abundance for genes associated with myeloid cell function. We conclude that by removing the confounding factor of anti-microbial activity, this has revealed for the first time the full breadth of this drug’s immunomodulatory potential within an *in vivo* inflammatory environment. These results may aid interpretation of the mode of action of AmBisome ^®^ in other settings, such as fungal disease.

Splenomegaly in experimental VL is associated with compartment specific remodelling of the red and white pulp, mediated through the action of TNF, inflammatory monocytes and loss of stromal cell integrity ^32, 49– 52^. Analysis of the splenic response at R _x_+7 provided evidence for diminution of the cellular responses though not to the same extent as seen in the liver. Notably, examination of residual genes DE in treated vs. naïve mice indicated that these reflected a number of pathways associated with remodelling and stem cell activity. This is perhaps not surprising given that these mice still exhibit splenomegaly and architectural disruption. Spleen structure is highly dynamic during infection, with the capacity for rapid resolution once infectious load has been cleared. For example, in the case of murine cytomegalovirus infection, spleen architecture and function is fully restored within days of the drop in tissue viral load, through mechanisms that involve the reactivation of RORγ-dependent lymphoid tissue inducer cells ^53, 54^. In contrast, with *L. donovani* infection restoration of lymphoid tissue architecture by the receptor tyrosine kinase inhibitor sunitinib was observed to be independent of RORγ ^32^ and splenomegaly can persist even after highly effective elimination of parasite load.

Transcriptomic analysis of the spleen response to *L. donovani* infection highlighted the complexity and dynamics of this process ^30^, identifying key pathways with progressive evolution of a complex immune and metabolic environment. Our data indicate that much of this remains intact even after parasite clearance, with only some reduction in macrophage activation signatures. We cannot rule out, and it is indeed likely, that splenomegaly and a state of immune activation persists partly as a consequence of low-grade infection below the limits of detection by the smear technique used here. However, it is noteworthy that the presence of parasite and parasite-mediated immune responses are not in themselves the sole regulator of this process. For example, mice treated with sunitinib retain a full parasite load yet show restoration of splenic architecture and partially regain immune competence ^32^, indicating the independence of pathology and parasite load.

In the clinic, splenomegaly is a common diagnostic characteristic for VL. The extent of splenomegaly at presentation is a significant risk factor for treatment failure, as is the presence of splenomegaly at the end of standard of care. For example, a study in southern Sudan indicated an odds ratio for relapse of 5.50 (1.84, 16.49) for patients discharged with a Hackett grade for splenomegaly ≥ 3 ^29^. Our data go some way to understanding the ongoing process of splenic remodelling that may also therefore be occurring in human disease. Whether these would be accelerated, as in the mouse by the use of combination therapies targeting remodelling, such as sunitinib, remains to be seen. It would also be of interest to evaluate the response of mice to AmBisome ^®^ treatment after longer periods of recovery.

Although not directly analysing the tissue response, others have examined the response to treatment at the transcriptional level. Gardinassi *et al.* reported on a whole blood transcriptomic analysis comparing 8 patients admitted with *L. infantum* VL in Teresina, Brazil; 8 different patients in remission following treatment for 2–5 months with antimonials; 12 DTH-positive putative asymptomatics and 15 DTH-negative healthy endemic controls ^44^. Despite limitations in study design, these data suggest that blood transcriptional profiles of patients in regression are distinct from both active cases and healthy controls. More recently, Fakiola *et al.* conducted a longitudinal study of the whole blood transcriptomic signature in *L. donovani* VL patients in Bihar, India, examining two independent cohorts (n=10 and 11) of patients before and after different regimens of amphotericin B treatment (repeated non-liposomal amphotericin B and single dose AmBisome ^®^) ^20^. As noted here, the major pathways associated with treatment response were related to interferon signalling, immune response and myeloid cell function. More importantly in the context of the current discussion, PCA analysis clearly demonstrated that the whole blood transcriptome of treated and cured VL patients does not return to a homeostatic baseline, at least within the 30 day follow up period. The extent to which these responses normalised also varied between treatment regimens, suggesting that blood transcriptomic signature may be a powerful tool for identifying biomarkers associated with treatment success ^20^. Collectively, these data suggest that patients discharged after receiving treatment for VL are some way away from fully restoring normal immune and physiological functions. This may have significant implications for understanding the basis of VL relapse and progression to PKDL, as well as responses to secondary unrelated infections and / or vaccines. Further studies are in progress to address these issues in patients with VL in East Africa ( https://www.prevpkdl.eu).

In conclusion, the results presented in this manuscript have identified the key transcriptional changes associated with AmBisome ^®^-induced parasite clearance in the spleen and liver of BALB/c mice infected with *L. donovani* and the extent to which such parasitologically-cured mice still deviate from homeostasis. In addition, we have identified previously hidden *in vivo* responses associated with administration of AmBisome ^®^. Together, these data provide the foundation for further studies on tissue level transcriptional responses to treatment in humans and a rich resource for scientists wishing to further explore the mode of action of AmBisome ^®^.

## Data availability

### Underlying data

Tissue and host species specific transcriptional changes in models of experimental visceral leishmaniasis, Mus musculus, Microarray data, Ascension number GSE113376: https://www.ncbi.nlm.nih.gov/geo/query/acc.cgi?acc=GSE113376

Tissue-specific transcriptomic changes associated with AmBisome treatment of mice with experimental visceral leishmaniasis [LV9], Mus musculus, Microarray data, Ascension number GSE140799: https://www.ncbi.nlm.nih.gov/geo/query/acc.cgi?acc=GSE140799

Tissue-specific transcriptomic changes associated with AmBisome treatment of mice with experimental visceral leishmaniasis, Mus musculus, RNA-Seq data, Ascension number GSE138825: https://www.ncbi.nlm.nih.gov/geo/query/acc.cgi?acc=GSE138825

Whole slide images and individual mouse metadata will be available from www.leishpathnet.org (study designations CRACKIT-1 and CRACKIT-2). Requests for access to tissue samples from these studies will be accommodated where possible and subject to availability.

### Extended data

Open Science Framework: CRACKIT Virtual Infectious Diseases project, https://doi.org/10.17605/OSF.IO/9WSDK ^55^. This version of the project registered on 22 ^nd^ November 2019.

This project contains the following extended data:

- **Figure S1. STRING analysis of down regulated genes in liver after AmBisome** ^®^ **treatment at R _x_+7.** GO terms related to host defense (red) immune system process (blue) and KEGG pathways cytokine-cytokine receptor pathways (green) and Leishmaniasis (yellow) are indicated.
- **Figure S2. STRING analysis of up regulated genes in liver after AmBisome** ^®^ **treatment at R _x_+7.** GO terms related to fatty acid metabolism (red), monocarboxylic acid metabolic processes (blue) and lipid metabolic process (light green) and KEGG pathways insulin signalling (dark green), biosynthesis of fatty acids (purple) and fatty acid elongation (yellow) are indicated.
- **Figure S3. STRING analysis of differentially expressed genes in liver after AmBisome ^®^ treatment of *M. bovis* BCG infected mice at R _x_+7.** Reference publications related to lysosomal and innate immunity (red; 9/17 genes; FDR 5.69x10 ^-12^; ^1^), meningiomas (blue; 19/93 genes; FDR 3.92x10 ^-11^; ^2^) CNS inflammation activation (light green; 14/39 genes; FDR 3.10x10 ^-10^; ^3^), lupus (yellow; 17/78 genes; FDR 3.10x10 ^-10^; ^4^) and atherogenesis (purple; 17/78 genes; FDR 3.10x10 ^-10^; ^5^) are indicated in footnote ^a^
- **Table S1. DE genes following AmBisome** ^®^ **treatment in spleen and liver of *L. donovani* infected BALB/c mice**. Each tab shows genes lists for each time point and each organ, with log2FC.
- **Table S2. GSEA analysis for liver and spleen DE genes in *L. donovani* infected mice at both times post AmBisome** ^®^ **treatment.** Relates to Figure 3. Note: no significant enrichments were found for spleen at d36.
- **Table S3. DE gene identified in the liver of AmBisome** ^®^ **treated BALB/c mice infected with *M. bovis* BCG.** DE list contains all genes passing p value cut off of 0.05 with no FC threshold.
- **Table S4. DE genes in spleen and liver of *L. donovani* infected mice, comparing AmBisome ^®^ treated mice to naïve mice.**
- **Table S5. Pathway analysis of residual DE genes in liver of AmBisome ^®^ treated mice *L. donovani* infected mice, compared to naïve mice.** Enrichments are shown for the following: GO Biological Processes, Molecular Function, Mouse Gene Atlas and WikiPathways2019Mouse.
- **Table S6. Pathway analysis of residual DE genes in spleen of AmBisome ^®^ treated mice *L. donovani* infected mice, compared to naïve mice.** Enrichments are shown for the following: GO Biological Processes, Molecular Function, Mouse Gene Atlas and WikiPathways2019Mouse.

Data are available under the terms of the Creative Commons Zero "No rights reserved" data waiver (CC0 1.0 Public domain dedication).

## Note


^a^ 1. Alam MS, Getz M, Safeukui I, Yi S, Tamez P, Shin J, *et al.* Genomic expression analyses reveal lysosomal, innate immunity proteins, as disease correlates in murine models of a lysosomal storage disorder. PloS one. 2012;7(10):e48273. Epub 2012/10/25. doi: 10.1371/journal.pone.0048273. PubMed PMID: 23094108; PubMed Central PMCID: PMCPMC3477142.2. Domingues PH, Teodosio C, Otero A, Sousa P, Ortiz J, Macias Mdel C, *et al.* Association between inflammatory infiltrates and isolated monosomy 22/del(22q) in meningiomas. PloS one. 2013;8(10):e74798. Epub 2013/10/08. doi: 10.1371/journal.pone.0074798. PubMed PMID: 24098347; PubMed Central PMCID: PMCPMC3788099.3. Bonasera SJ, Arikkath J, Boska MD, Chaudoin TR, DeKorver NW, Goulding EH, *et al.* Age-related changes in cerebellar and hypothalamic function accompany non-microglial immune gene expression, altered synapse organization, and excitatory amino acid neurotransmission deficits. Aging (Albany NY). 2016;8(9):2153-81. Epub 2016/10/01. doi: 10.18632/aging.101040. PubMed PMID: 27689748; PubMed Central PMCID: PMCPMC5076456.4. Berthier CC, Bethunaickan R, Gonzalez-Rivera T, Nair V, Ramanujam M, Zhang W, *et al.* Cross-species transcriptional network analysis defines shared inflammatory responses in murine and human lupus nephritis. J Immunol. 2012;189(2):988-1001. Epub 2012/06/23. doi: 10.4049/jimmunol.1103031. PubMed PMID: 22723521; PubMed Central PMCID: PMCPMC3392438.5. Ley K, Miller YI, Hedrick CC. Monocyte and macrophage dynamics during atherogenesis. Arterioscler Thromb Vasc Biol. 2011;31(7):1506-16. Epub 2011/06/17. doi: 10.1161/ATVBAHA.110.221127. PubMed PMID: 21677293; PubMed Central PMCID: PMCPMC3133596.
