# Supplementary material for: Tissue-specific transcriptomic changes associated with AmBisome® treatment of BALB/c mice with experimental visceral leishmaniasis
Source: Wellcome Open Res. 2019 Dec 10;4:198. [Version 1] doi: 10.12688/wellcomeopenres.15606.1 (PMC6961418; doi:10.12688/wellcomeopenres.15606.1)
Supplement: Peer review history

# Peer review: Reviewer response for version 1

**DOI:** 10.21956/wellcomeopenres.17094.r37346

**Published:** 2020-01-31

**Authors:** Abhay R. Satoskar

**Affiliations:** 1 Department of Pathology, Ohio State University Medical Center, Columbus, OH, USA

This paper analyzes the liver and spleen transcriptomes from *L. donovani *infected BALB/c mice temporally following Ambiosome treatment. The key message is that liver and spleen responses to Ambiosome treatment are distinct and they are not restored after parasitological cure. Overall, the paper is well written with data supporting the conclusions. The information could help in better elucidation of the host factors that may be responsible for Ambiosome treatment failures reported in certain endemic countries. The only weakness is that the study is descriptive and not mechanistic. Did authors experimentally validate any of the transcriptome data? It is also possible mouse genetic background could influence transcriptome profile during *L. donovani *infection. This would be interesting to determine. Such studies would be useful but may be beyond the scope of the present paper, which is important for the field.

I confirm that I have read this submission and believe that I have an appropriate level of expertise to confirm that it is of an acceptable scientific standard.

# Peer review: Reviewer response for version 1

**DOI:** 10.21956/wellcomeopenres.17094.r37344

**Published:** 2020-01-14

**Authors:** Syamal Roy

**Affiliations:** 1 National Institute of Pharmaceutical Education & Research, Kolkata, India

This manuscript presents a study on the tissue-specific response of AmBisome® treatment in BALB/c mice infected with experimental visceral leishmaniasis. The investigators made a comparative analysis of differential expression of genes in spleen and liver of the drug -treated vs untreated *L. donovani-*infected BALB/c mice at day 36 (Rx +1) and day 42 (Rx +7), using various statistical techniques, quantitative morphometry and pathway enrichment analyses. The study indicates that though the parasite burden in both spleen and liver gets cleared by Rx +7, the pattern of cure follows distinct tissue-specific kinetics, as reflected by significant variations in number of up and down regulated genes in spleen and liver of AmBisome®-treated vs. untreated mice. GO enrichment analysis was carried out to find out the pathways, plausible enrichment of which might account for the observed variations in transcriptomic profiles of host liver and spleen following AmBisome® treatment. Using quantitative image analysis, authors attempted to establish that the changes in the liver transcriptomic profile associated with AmBisome® treatment might reflect cessation of granulomatous inflammation following parasite clearance. PCA analysis was carried out to evaluate tissue-specific divergences in the extent of restoration to homeostasis, while a binary cluster analysis along with heat-map representation was provided to establish restoration of 26 signature genes of *L. donovani *infection following drug treatment. The investigators also used an infection model with *M. bovis* BCG to examine the direct effects of AmBisome® treatment, if any, on host transcriptome, only to find some subtle effects. In a nutshell, the study presented in the manuscript might be helpful in understanding tissue specific transcriptional responses in host to AmBisome® treatment in case of *L.donovani* infection.

 However, there are some small queries:

1. In PCA analysis, in case of spleen (Fig 6A), the treated d42 mice appear at the most distant position wrt other samples along Dim1. Why? In case of liver (Fig 6B), untreated and treated D42 samples are much closer to one another along Dim1, as compared to other samples. What could be its plausible reason? Since Dim1 explains more than 60% and 70% variations in these two cases, these questions should have been addressed.
2. In Venn diagram, (Fig 7), there are 213 genes, which seem to be differentially expressed in all d36 and d42 samples as compared to naïve ones. What are these genes that seem to get affected upon infection, and remain affected in both spleen and liver in D42?
3. In cluster analysis (Fig 8), both for liver and spleen samples, D42 and D36 treated and untreated samples are jumbled up within themselves under a distinct node (separate from naïve samples). What does that indicate for signature genes?

I would, therefore, recommend acceptance of the manuscript with minor revision (addressing the queries made above).

I confirm that I have read this submission and believe that I have an appropriate level of expertise to confirm that it is of an acceptable scientific standard.

# Peer review: Reviewer response for version 1

**DOI:** 10.21956/wellcomeopenres.17094.r37347

**Published:** 2019-12-27

**Authors:** Yasuyuki Goto

**Affiliations:** 1 Laboratory of Molecular Immunology, Graduate School of Agricultural and Life Sciences, The University of Tokyo, Tokyo, Japan

AmBisome has been used as the first line therapy for VL and exploring host responses to the treatment is important to understand the drug’s mode of action and potential adverse events brought by the treatment. The authors addressed such the treatment-associated host responses using a transcriptomic approach. The data presented are sufficient and analyses of those data are also comprehensive.

 There are some comments which may facilitate to improve the article:

1. Interpretation of data on post-treatment transcriptome is condensed in ‘not back to normal yet but in the middle of inflammatory condition (no treatment) and no inflammation (no infection)’. It is probably the most important finding but the authors are encouraged to discuss more on genes uniquely up- or down-regulated in AmB-treated mice, because those might be involved in parasite clearance mechanism as well as adverse events by the drug. In fact, PCA shown in Figure 6 also implies unique responses in treated groups.
2. Figure 3 also needs more discussions. For example, ‘UP in memory CD8 T cells’ and ‘Down in CD4 T cell expansion’ include genes both up-regulated and down-regulated in the liver at D7 of treatment. What are actual ‘up’ and ‘down’ genes in the categories and how do both ‘up’ and ‘down’ genes explain the successful clearance of parasites in the liver? I believe this part is also important to understand the mode of action of AmBisome in VL treatment.

 Also, some minor comments:

1. Log2: 2 better to be subscript.
2. Page 4, Paragraph ‘Tissue transcriptomics’: ‘DE expressed’ should be just ‘DE’.
3. Figure 2: PPR should be PRR.
4. Figure 5B: Please include data on uninfected mice so that the readers can see hepatomegaly.
5. Figure 7: Because this figure only describes infected-treated groups but not the infected-untreated, Rx+1/Rx+7 are more appropriate than d36/d42.

I confirm that I have read this submission and believe that I have an appropriate level of expertise to confirm that it is of an acceptable scientific standard.
